# Effective Schottky barrier lowering of NiGe/p-Ge(100) using Terbium interlayer structure for high performance p-type MOSFETs

**DOI:** 10.1038/s41598-020-61011-4

**Published:** 2020-03-04

**Authors:** Sunil Babu Eadi, Jeong Chan Lee, Hyeong-Sub Song, Jungwoo Oh, Ga-Won Lee, Hi-Deok Lee

**Affiliations:** 1 Department of Electronics Engineering, Chungnam National University, Daejeon, Korea; 2 School of Integrated Technology, Yonsei Institute of Convergence, Technology, Yonsei University, Incheon, Korea

**Keywords:** Electrical and electronic engineering, Nanoscale devices, Nanoscale materials

## Abstract

Ultra-low contact resistance at the interface between NiGe and p-Ge, i.e., NiGe/p-Ge was achieved by introducing terbium (Tb) as an interlayer in forming NiGe using Tb/Ni/TiN structure. The contact resistance value obtained using the circular transmission line model for an 8-nm thick Tb interlayer sample was 7.21 × 10^−8^ Ω·cm^2^, which is two orders of magnitude less than that of reference sample (without the Tb interlayer) of 7.36 × 10^−6^ Ω·cm^2^. The current–voltage characteristics were studied at a temperature range of −110 ~ 25 °C to determine the effective Schottky barrier height (eSBH). An eSBH of 0.016 eV was obtained for the 8-nm thick Tb interlayer. Various Tb interlayer thicknesses were selected to study their effect on the contact resistance. The Tb interlayer surface and structural properties were characterized using FESEM, XRD, XPS, TEM, and SIMS analyses.

## Introduction

To harness the benefits of higher carrier mobilities materials such as Germanium (Ge), III-V group compounds, and two dimensional materials in advanced complementary metal-oxide semiconductor (CMOS); devices integration and processing techniques have to well established for making high performance devices^1–5^. Among higher carrier mobilities materials Ge, in particular has received ever increasing attention owing to its excellent electron and hole mobilities compared with those of silicon (Si)^6^. Utilizing Ge as a channel material has several advantages. First, Ge enables complementary metal-oxide semiconductor (CMOS) transistors in a single material framework, thus significantly simplifying the manufacturing process like silicon. Second, as Ge and Si are both IV group elements, Ge is compatible with current manufacturing technologies and industrial facilities. Besides, since the Fermi level of metal/Ge contact lies close to the Ge valence band, a low hole SBH will result in very appropriate Ge pMOSFET, with improved electrical characteristics^7,8^. Hence, it is important to study the various aspects of Ge transistor processes and device characteristics. Primarily, Source/ Drain (S/D) and Gate Contact resistance and Thermal stability of Ge based CMOS have to be studied. Also, with downscaling of transistor size it is expected that the contact resistance between the contact material and the Ge substrate will be more dominating in future sub-5 nm CMOS technology^9^. So, these issues must be quickly addressed to improve the performance of Ge MOSFETs.

Recently, many researchers as shown that the Metal-germanide (M-Ge) contacts similar to self-aligned metal-silicide technique generally used for Source/Drain (S/D) and gate contacts in Si can be very useful^10^. Among many metal-Germanides studies, Nickel germanide (NiGe) show superior contact properties owing to its low resistivity and shallow junction formation^11,12^. However, NiGe contacts still faces two major drawbacks i.e., (i) thermal instability above 500 °C due to formation of agglomeration, and (ii) contact resistance still far from ideal resistance of below 10^−9^, for making fully operational Ge based device technology. In this regard, scientists have shown that, addition of a new interlayer beneath the NiGe contact could help in increasing in the thermal stability and decreasing in contact resistance by Schottky barrier height (SBH) modulation at the same time. Jablonka *et al*., reported the morphological and thermal stability of NiGe enhanced by using Tantalum (Ta) and Tungsten (W) metal interlayers^13^. Liew *et al*., and Lee *et al*., groups have studied the thermal ability of NiGe films using Zirconium (Zr) interlayer and reported the delayed in agglomeration beyond 600 and 550 °C respectively^14,15^. Zhu *et al*., shown that using titanium (Ti) interlayer can enhance the thermal stability of NiGe by acting as capping layer and prevent agglomeration at elevated temperature^16^. However, many of this transition metal having high work function, not helpful in reducing the contact resistance in p-Ge for device fabrication use even though thermal stability improves. Therefore, using interlayer materials which could help in improve the thermal stability and achieve low contact resistance are essential. In this regard, using Rare Earth Metals (REMs) as an interlayer in NiGe/Ge junctions for reduction of contact resistance and thermal stability could be beneficial. It is well known that REMs with lower work function are best candidates for reducing contact resistance in NiGe/p-Ge junction by eSBH modulation. Many reports have shown that, REMs owing to their low SBH values and similar lattice parameters to Si have been applied in microelectronics for very long time^17–19^. Demeulemeester *et al.*, studied the Ni-REMs (Y, Gd. Dy and Er) Silicides formation properties and their effect on SBH modulation^20^. Ishikawa *et al.*, has shown the decrease in work function of PtSi from 4.92 to 4.57 eV using Yb interlayer^21^. Our previous work on NiSi/Si with ytterbium (Yb) has shown that 0.15–0.38 eV work function reduction^22^. It’s very important to explore REMs interlayers in NiGe/Ge junctions.

In this paper, we report first time the effect of Tb interlayer in NiGe/p-Ge junction. The role of terbium as an interlayer for reduction of NiGe/p-Ge interface contact resistance and modulation of eSBH in NiGe/p-Ge were investigated. NiGe/p-Ge with Tb interlayer was fabricated and the specific contact resistivity was obtained using the circular transmission line model (CTLM) patterning process^23^. The eSBH was obtained from the current–voltage measurement at various temperatures. The effect of the Tb interlayer with a split of its thickness on the contact resistance at the interface was systematically analysed and thermal stability of the formed NiGe with and without Tb were also studied.

### Experiment procedure

**Figure 1:**
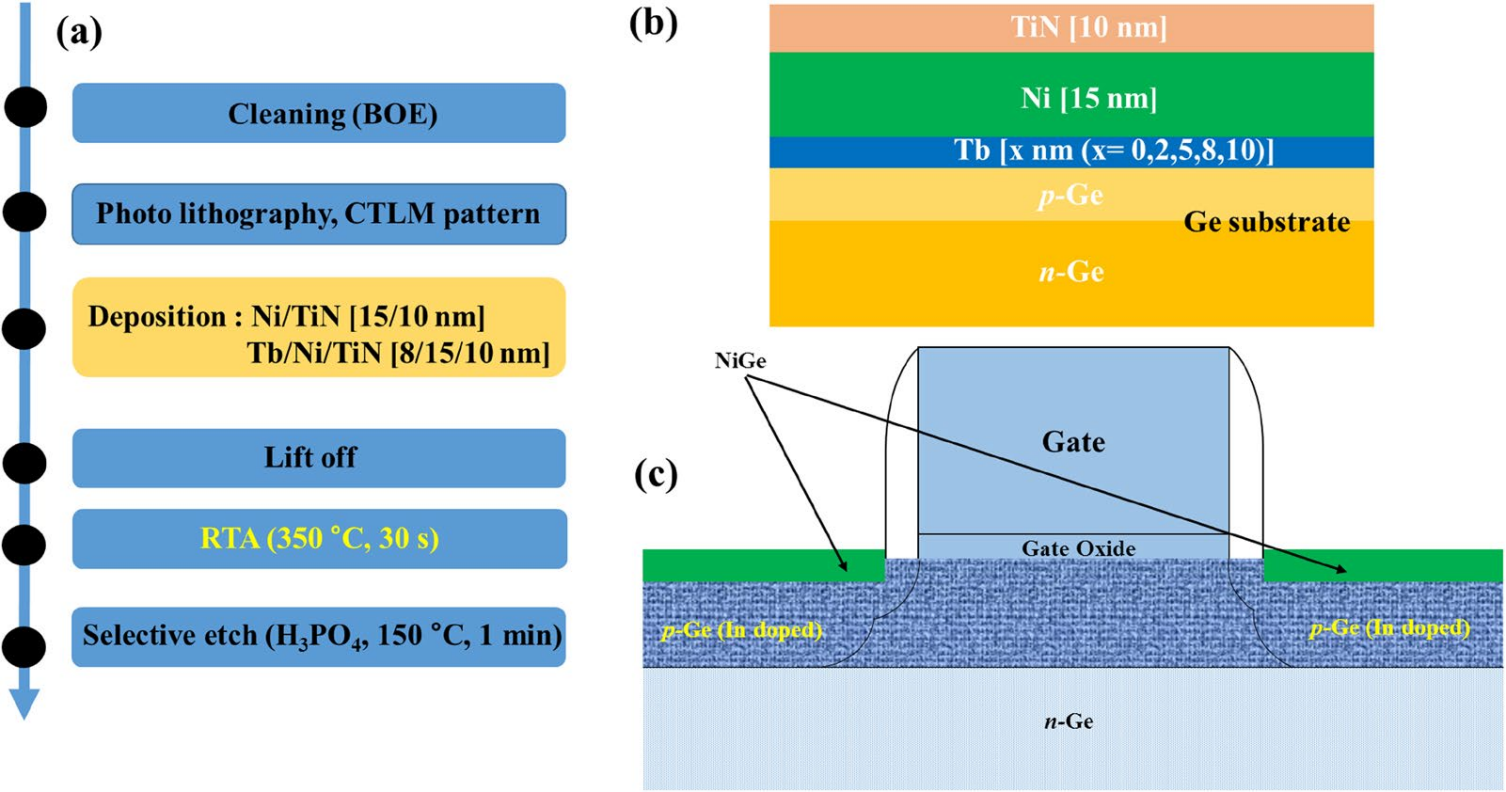
(**a**) Fabrication flow chart of NiGe on p-Ge substrate with and without Tb interlayer. (**b**) Schematic diagram of interlayer structure deposition on Ge substrate and (**c**) Schematic diagram of NiGe contacts formed on the MOSFET Source/Drain (S/D) regions.

Arsenic doped Ge (100) wafers were used as a substrates. Indium (In) was used as a p- type dopant by ion implantation with a dose of 5 × 10^14^ cm^−2^ at 50 keV. The dopant activation of In-implanted Ge wafers was performed at 550 °C for 10 s using an RTA system. After activation the substrate was cleaned using buffered oxide etchant (BOE) for 30 s. Next, Tb/Ni/TiN (2, 5, 8, 10/15/10 nm) films were deposited *in situ* using radio-frequency (RF) magnetron sputtering on the In-doped p-type Ge substrates. Ni/TiN (15/10 nm) structure without Tb is also formed as a reference sample for comparison. The TiN layer was deposited as capping layer on top to prevent the oxidation of Ni during RTA process for the formation of NiGe. NiGe was formed by loading it into the RTA annealing chamber and annealed for 30 s at 350 °C. Finally, the unreacted Ni and TiN was etched using H_3_PO_4_ solution at 150 °C for 1 min. Schematic diagrams of Interlayer stack structure and CTLM pattern fabrication on Germanium substrate are shown in Fig. 1.

## Results and Discussion

**Figure 2:**
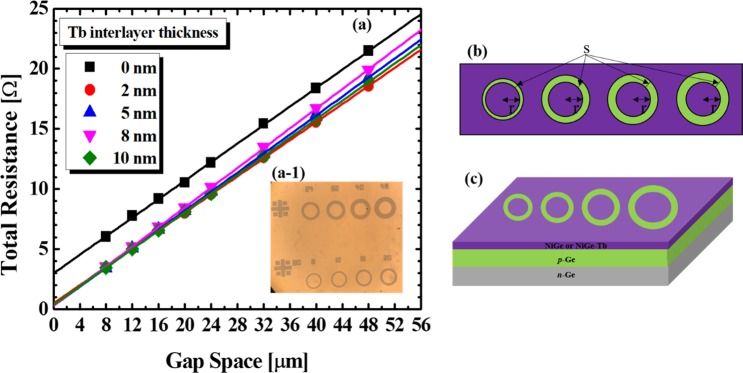
(**a**) Plot of total resistance versus Gap space of CTLM pattern for extraction of specific contact resistivity with different Tb interlayer. (**b**,**c**) CTLM denotation r (80 µm) is radius of inner circle, and s is radius difference between inner and outer circle (gap space ~8, 12, 16, 20, 24, 32, 40, 48 µm) of NiGe on Ge substrate.

The specific contact resistance is an imperative parameter impacting the interfacial properties of the connected (NiGe/p-Ge and NiGe-Tb/p-Ge) metallization frameworks. An appropriate way is to measure electrical properties quantitatively, to obtain specific contact resistance ρ_c_ (Ω·cm^2^), from which the contact resistance (R_c_) can be determined. Fig. 2 shows the contact resistance after introducing the Tb interlayer at the NiGe/p-Ge interface. The total resistance (R_T_) was measured via kelvin four-probe method, and ρ_c_ was obtained from Sheet Resistance (Rsh) and Transfer Length (L_T_) by using eq (1) ^24,25^:

1 $${{\boldsymbol{\rho }}}_{{\boldsymbol{c}}}={{\boldsymbol{R}}}_{{\boldsymbol{sh}}}{{\boldsymbol{L}}}_{{\boldsymbol{T}}}^{2}$$

The ρ_c_ value of the reference sample, i.e. NiGe/p-Ge obtained from the CTLM patterning process, is 7.36 × 10^−6^ Ω cm^2^, and the ρ_c_ values of the samples with Tb interlayer thicknesses of 2, 5, 8, and 10 nm are 2.15 × 10^−7^, 6.61 × 10^−8^, 7.21 × 10^−8^, and 1.17 × 10^−7^ Ω·cm^2^, respectively. The results show that the Tb interlayer helps reduce the contact resistance at the NiGe/p-Ge interface by a factor of 100 and considerably lower than the previous reports^26–28^. Although the resistances of both the 5 nm and 8 nm-thick Tb samples were low, we selected the latter to find the reason behind the low contact resistance.

**Figure 3:**
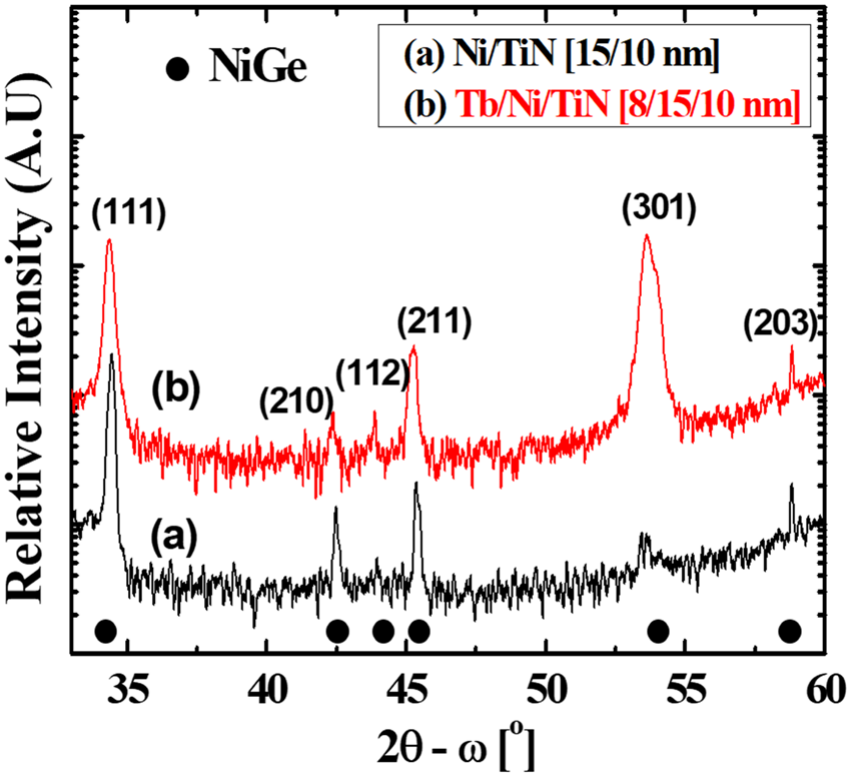
X-ray Diffraction profile of NiGe samples with and without Tb interlayer.

First, to confirm the formation of the NiGe phase in the reference sample and Tb interlayer sample during the RTA process, X-ray diffraction (XRD) was performed on the samples. The XRD plots of two samples are shown in Fig. 3. The peaks corresponding to NiGe phase can be seen in Fig. 3(a). The major peaks can be indexed to the NiGe crystalline structure and NiGe (111), (210), (211) peaks are clearly visible^29^. Fig. 3(b) shows the XRD peaks of NiGe with the Tb interlayer. In Tb sample, we notice reduction of intensities of NiGe peak and additional peak at 54° corresponding to (301) peak of NiGe is noticed in the presence of Tb. This concludes that the incorporation of Tb within the NiGe film changes crystalline orientation of NiGe on the substrate. A new intermetallic phase with preferred crystalline orientations, such as (112) and (301), may favour a stable germanidation formation, which could reduce the contact resistance and make a stable germanide.

**Figure 4:**
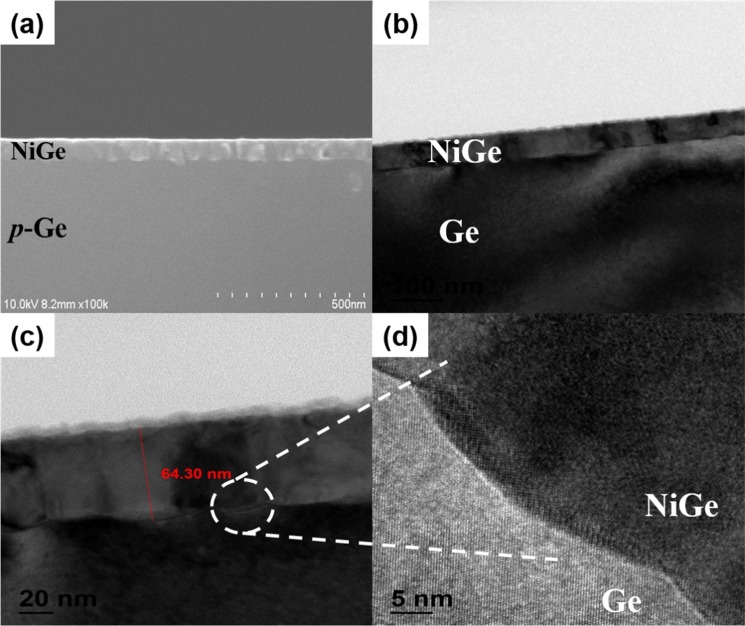
(**a**) FESEM image of NiGe formation on Ge substrate. (**b**,**c**) TEM images of NiGe formation, (**b**,**c**) high magnification image of NiGe, and (**d**) NiGe/p-Ge interface showing the snow-plow shape.

Further, TEM images of NiGe and NiGe-Tb samples were taken. Fig. 4(a–d) show the TEM images of reference NiGe. Fig. 4(a) shows the cross-sectional FESEM image of NiGe layer on the Ge substrate. The images show a uniform formation of the NiGe film on the p-Ge substrate following the RTA process. The TEM images clearly shows the formation of NiGe and the average thickness of NiGe measured to be ~64 nm and it was noticed that the formed NiGe exhibits a snow-plow shape at the interface because of the grooving effect into substrate Ge^30–32^. The mechanism related to effect of RTA temperature and Ni film thickness in the formation of NiGe is well reported by various research groups. Lee *et al*., reported, fully formed NiGe phase generally starts with Ni-rich germanide phase starting at 250 °C and with increasing annealing temperature from 300–500 °C Ni is fully consumed to form highly textured NiGe film^33^. Also the rate of formation of NiGe depends on the initial thickness of the Ni film deposited as studied by Zhu *et al*., and for x nm thickness of Ni film; approximately ~2.5–3 times x nm NiGe is formed^34^.

**Figure 5:**
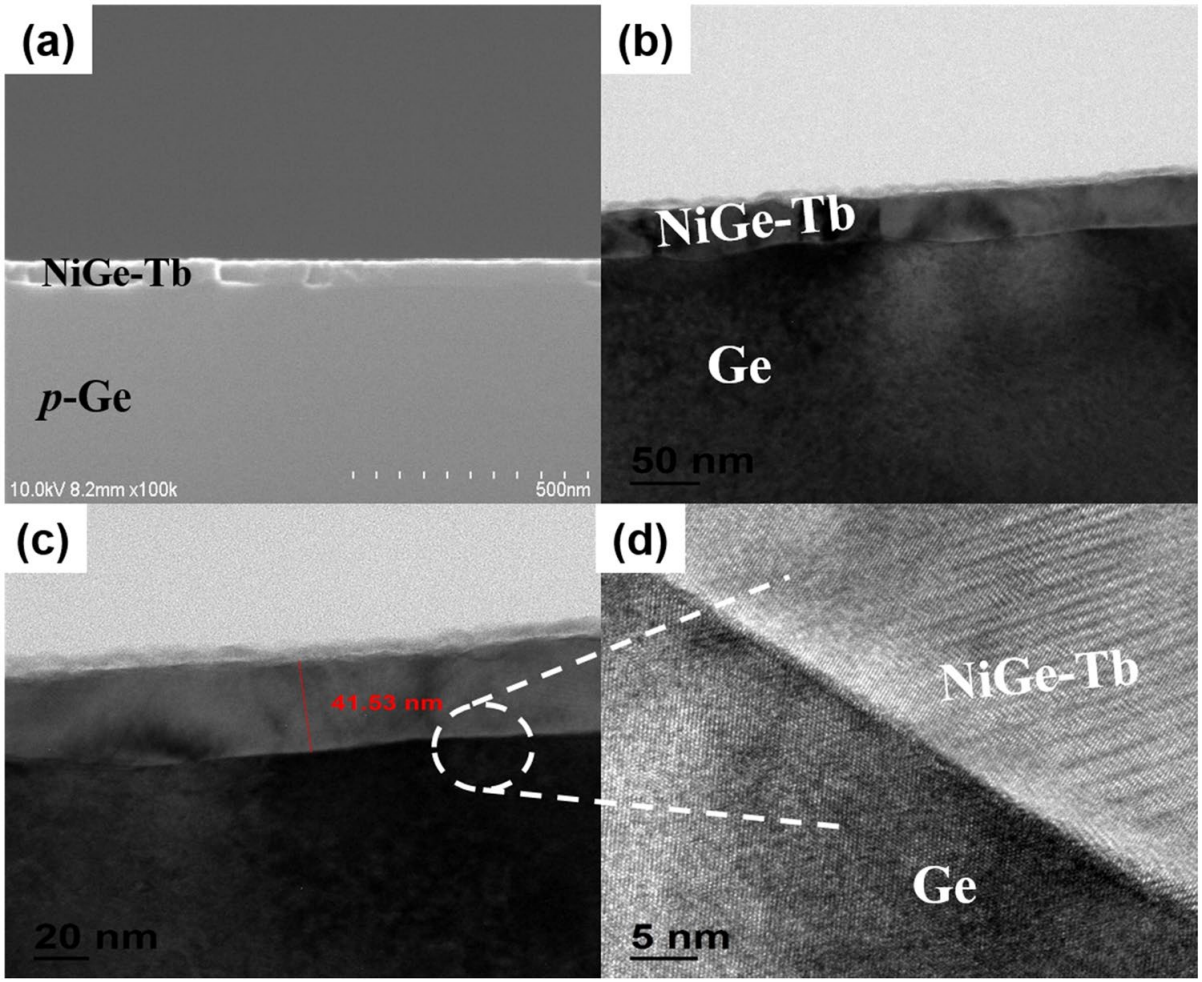
(**a**) FESEM image of NiGe formation on Ge substrate. (**b**,**c**) TEM images of NiGe formation, (**b**,**c**) high magnification image of NiGe, and (**d**) NiGe/p-Ge with unifrom interface.

Fig. 5(a–d) show the TEM images of NiGe with Tb interlayer with a thickness of 8 nm. Fig. 5(a) shows the cross-sectional FESEM image of NiGe-Tb/p-Ge. Here it is noticed that, by applying Tb interlayer the thickness of the NiGe reduced. The average thicknesses of NiGe with the Tb interlayer were measured to be 46 nm. The formed NiGe exhibits more uniform interface as compared to reference sample (NiGe). By comparing the NiGe thickness from TEM images of reference and Tb interlayer shows that, Tb interlayer effect hinders the diffusion of Ni into the Ge substrate^35,36^. By Considering the NiGe thickness formed with and without Tb interlayer, new insight’s in our experiment shows that, interlayer is essential for NiGe formation for uniform formation of NiGe contact.

**Figure 6:**
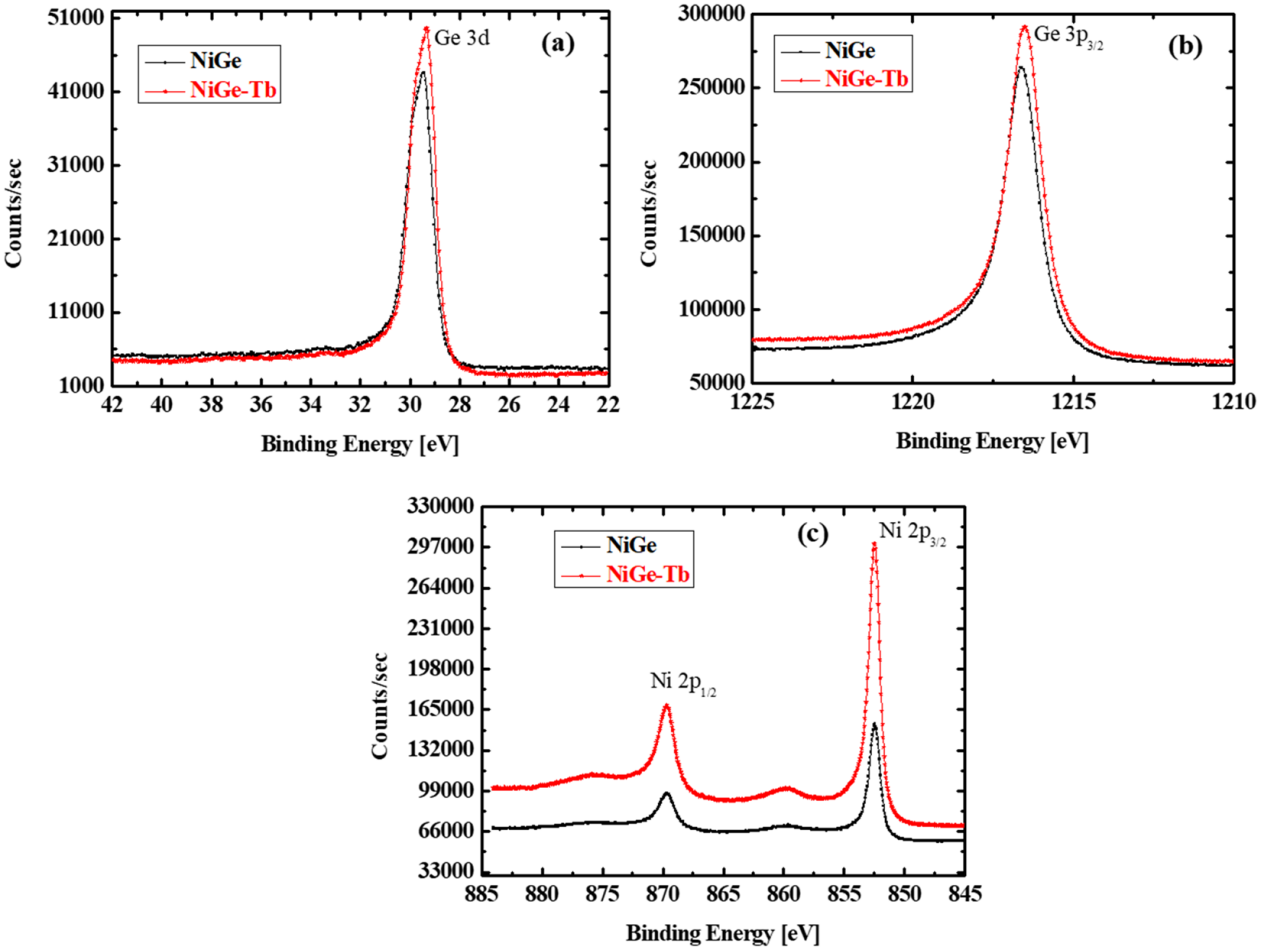
XPS spectral images of NiGe and NiGe with and without Tb interlayer., (**a**) Ge 3d, (**b**) Ge 3p_3/2_, and (**c**) Ni 2p_3/2_.

To confirm the NiGe formation and the effect of Tb interlayer, XPS studies were performed on NiGe and NiGe-Tb on Ge (001) samples obtained after RTA process as shown in Fig. 6. The evolution of Ni 2p3/2, Ge 2p3/2, and Ge 3d XPS peaks can be clearly seen in the spectrum Fig. 6. From Fig. 6(a,b) we can notice that, peaks of Ge 2p3/2 and Ge 3d and Ni 2p appearing at 29.45, 1216.6 and 852.40 cm^−1^ respectively. In the case of NiGe sample, the peaks can be attributed to Ni diffusion into bulk of Ge substrate and formation of NiGe after RTA. The atomic % ratio of Ni/Ge is found to be 1:2, indicating the Ni diffusion into Ge substrate. In NiGe-Tb samples the peaks Ge 2p3/2 and Ge 3d and Ni 2p show increase in intensities and right shift which attribute to higher binding energy Ge 2p3/2 and Ge 3d core level. In the case of Ni 2p3/2 the peak intensity increase, is due to higher exposed Ni surface area and increase in the nearest Ni-Ni distance due to insertion of Tb as previously reported^37,38^. Also chemical state of Tb in the NiGe-Tb alloy could be +3, +4 corresponding to Tb 4d peak according to pervious studied^39,40^. However we notice high background noise with no characteristic Tb 4d peaks was observed in the XPS spectrum, which could be due to low concentration of Tb in the sample. Further investigated is required for clear analysis. The atomic % ratio of Ni/Ge with Tb interlayer found to be 1:1. The ratio indicate the uniform formation of NiGe layer with controlled diffusion.

**Figure 7:**
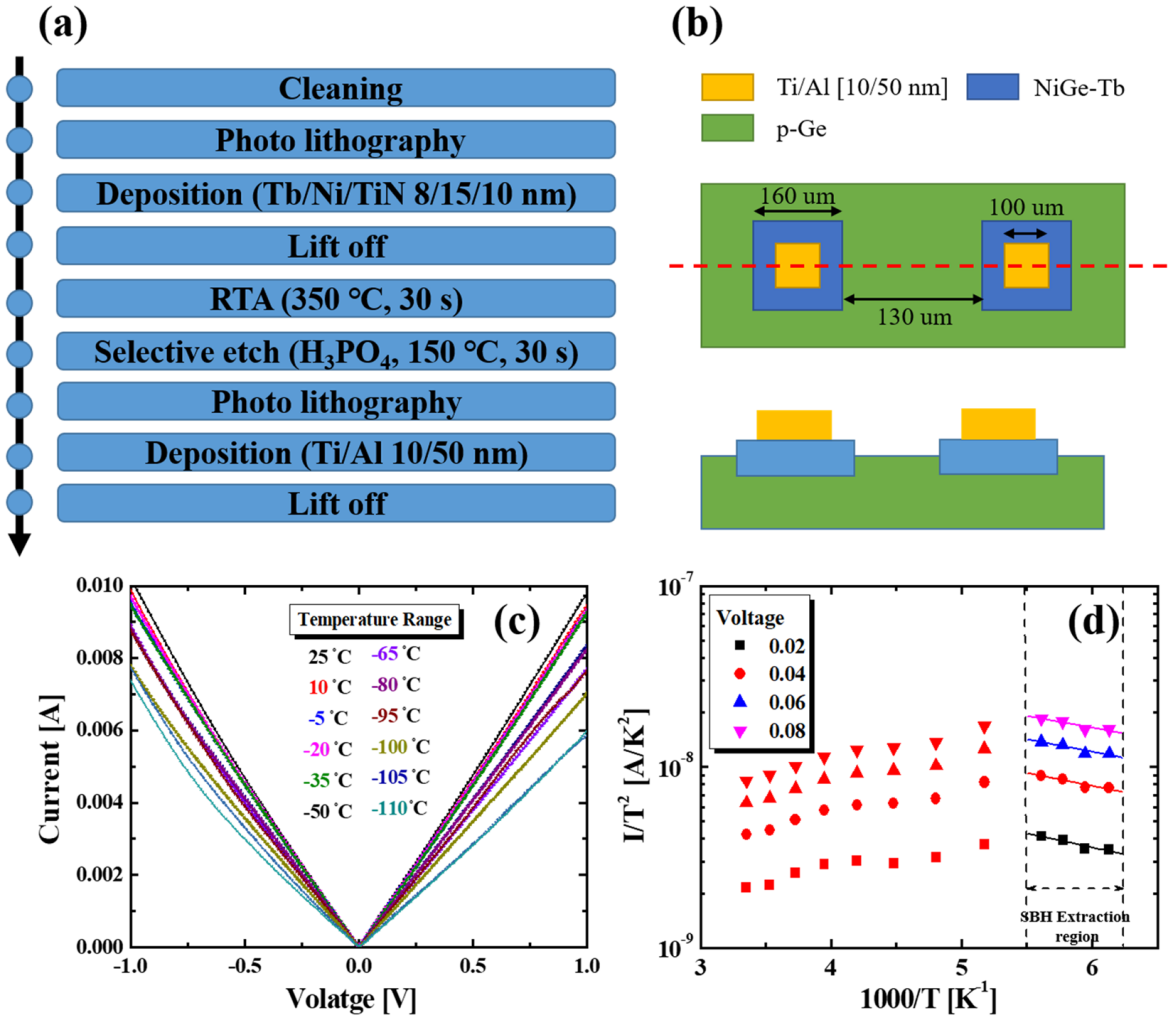
(**a**) Shows the flow diagram of diode fabrication for the SBH extraction, (**b**) schematics of the diode formed, (**c**) I–V characteristics of the 50 nm NiGe/Ge diode with 8 nm thick Tb Interlayer and (**d**) the reverse Arrhenius plots of a 50 nm NiGe on Ge with Tb segregation with a temperature variation from −110 to 25 °C.

**Figure 8:**
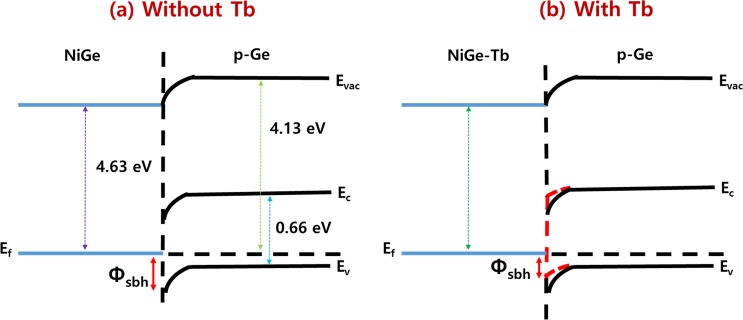
Schematic diagram of energy band of NiGe/p-Ge with and without Tb interlayer.

To investigate the effect of Tb interlayer during germanidation on the modulation of the eSBH, a diode was fabricated with Ti/Al [10/50 nm] contact PAD on NiGe-Tb (8 nm)/p-Ge sample. The fabrication flow chart and schematic diagram of the diode are shown in Fig. 7(a,b). Figure 7(c) shows the current–voltage (I–V) characteristics of the diode with the 8 nm-thick Tb interlayer in the temperature range of −110~25 °C with split of 15 °C. The germanidation temperature was 350 °C. The I–V characteristics exhibit Ohmic like properties of NiGe/p-Ge due to the very low effective Schottky barrier. The reverse current decreases with the decrease in the temperature, indicating the increase in the eSBH. The eSBH was calculated based on the thermionic emission theory^41,42^. The eSBH is determined from the slope of the curves in the low-temperature region of the Arrhenius plots as shown in Fig. 7(d). It is I/T^2^ versus 1/T plot at forward biases of 0.02, 0.04, 0.06, and 0.08 V and where the absolute temperature in Kelvin. The average eSBH was found to be 0.016 eV, which is considerably low for NiGe/n/p-Ge junctions than the previous reports^43–46^. Fig. 8(a,b) shows the schematics of energy band diagram of NiGe/p-Ge junction with and without Tb interlayer. Previous study has shown that, the work function of NiGe is about 4.63 eV^47^. In this study, after introducing Tb interlayer, the new interlayer work function increased thereby reducing the eSBH of the p-Ge. Therefore, the work function is altered by adding Tb metal in to NiGe.

**Figure 9:**
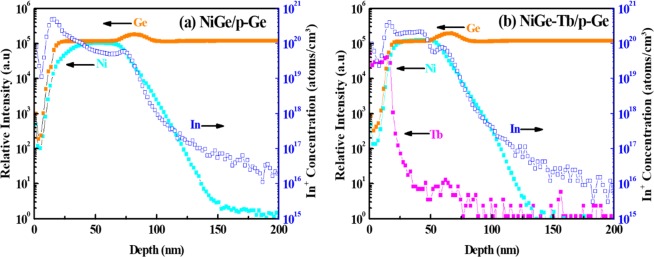
SIMS profile of ingredients for (**a**) NiGe without Tb and (**b**) NiGe with Tb interlayer.

Fig. 9(a,b) Shows the measured elemental depth profile using SIMS. The presence of Ge, Ni, and In elements can be confirmed in Fig. 9(a). The plot shows the Indiums atoms diffused throughout the Ge substrate and pile-up at the interface. The NiGe formation range can be estimated to around 65–70 nm, where the intensity of Ni decreases gradually. Fig. 9(b) confirms the presence of Tb interlayer in NiGe and its effect on the In distribution in the sample. In this case, the pile-up of In dopant increased at the interface of NiGe and p-Ge along with Tb pile-up and intensity diminish into the NiGe layer. This implies that both the tunnelling width and the eSBH could reduce due to the Tb incorporation. As mentioned earlier, low work function (3 eV) of Tb could lower the electron SBH and Tb atoms, with oxidation numbers of +3, +4 states, act like positive charges at the Ge side of the interface and assist in the hopping of the holes at the interface there by reducing the contact resistance drastically.

**Figure 10:**
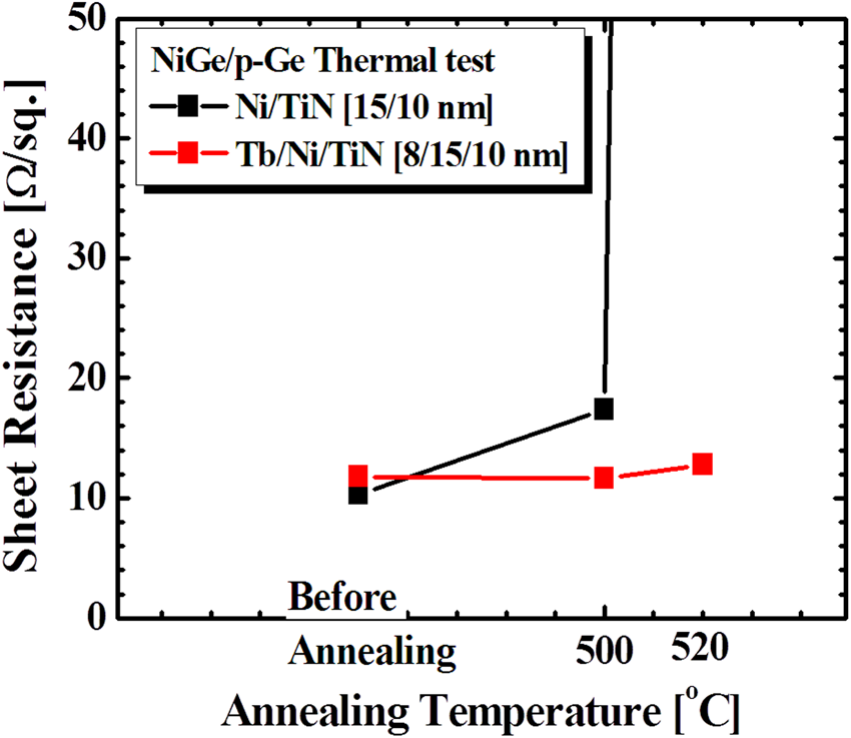
Sheet resistance verse Annealing temperature plot of Terbium interlayer sample in comparison to reference sample on Ge substrate.

Finally, thermal stability of NiGe with and without Tb interlayer are studied by annealing the samples for 30 min in thermal oven with N_2_ atmosphere. The thermal stability was analysed by measuring the sheet resistance of the annealed samples. Table 1 shows the sheet resistance values before and after annealing at 500 °C and 520 °C respectively. It can be noticed that for reference sample NiGe, sheet resistance gradually increases with annealing temperature 500 °C from 10.3 to 17.42 Ω/sq. and drastic change occur at 520 °C as shown in Fig. 10. The sheet resistance shoot to 509.4 Ω/sq., indicating instability due to agglomeration of NiGe. In the case, of NiGe with Tb interlayer, the sheet resistance remains stable even after increasing temperature to 520 °C. This confirms, Tb interlayer hinders the agglomeration by slowing the diffusion of Ni into Ge substrate.

**Table 1:** Sheet resistance values at different annealing temperature for Tb interlayer and reference samples.

Samples	Tb thickness [nm]	Before annealed	500 °C	520 °C
		**Sheet Resistance [Ω/sq.]**		
Ni/TiN	0	10.36	17.42	509.40
Tb/Ni/TiN	8	11.81	11.69	12.80

Thus, we anticipate that the development of a NiGe-Tb alloy compound with a low metal fermi level and enhanced dopant segregation close to the interface of NiGe/p-Ge are responsible for the decrease in the specific contact resistivity and increase the thermal stability of NiGe contacts.

## Conclusions

Demonstration of eSBH modulation of NiGe on p-Ge by Tb segregation was studied. Using Tb interlayer thickness of 8 nm, we achieved an extremely low eSBH of 0.016 eV, which is promising for p-MOSFETs, particularly for multiple-gate transistors and FinFETs with narrow fin widths. We discussed a possible mechanism explaining why the eSBH decreases with the increase in the Tb concentration at the NiGe/p-Ge interface. We speculate that the thinning of the Schottky barrier width at the NiGe/p-Ge interface is due to the pileup of Tb atoms at the Ge side, which reduced the effective hole SBH. These results can serve as a basis for developing high-performance transistors in the near future.
